# Immunological Markers of Cardiovascular Pathology in Older Patients

**DOI:** 10.3390/biomedicines13061392

**Published:** 2025-06-06

**Authors:** Akbota Bugibayeva, Almagul Kurmanova, Kuat Abzaliyev, Symbat Abzaliyeva, Gaukhar Kurmanova, Diana Sundetova, Merei Abdykassymova, Raushan Bitemirova, Ulzas Sagalbayeva, Karashash Absatarova, Madina Suleimenova

**Affiliations:** 1 Faculty of Medicine and Healthcare, Al-Farabi Kazakh National University, Almaty 050040, Kazakhstan; bota_88.20@mail.ru (A.B.); abzaliev_kuat@mail.ru (K.A.); abzaliyeva.symbat@gmail.com (S.A.); gaukhar.kurmanova@kaznu.kz (G.K.);; 2 Faculty of Information Technology, Al-Farabi Kazakh National University, Almaty 050040, Kazakhstan

**Keywords:** immunophenotyping, monocytes, cytotoxic lymphocytes, cytokines, cardiovascular diseases, immunosenescence

## Abstract

**Background:** The aging process is accompanied by changes in the immunological status of a person. Immunosenescence is considered a significant cause of the development of cardiovascular diseases (CVD) in elderly people. However, to date, the relationship between immune/inflammatory processes and diseases associated with age is considered quite complex and is not fully understood. Immunophenotyping and the intracellular production of cytokines involved in the processes of inflammatory aging will allow us to identify biomarkers that are associated with cardiovascular diseases in the elderly. **Objectives:** To identify immunological markers associated with the process of inflammatory aging in older individuals with cardiovascular diseases. **Methods:** CD-phenotyping and intracellular cytokine analysis of peripheral blood using the flow cytometry method were conducted in 52 people over 60 years of age (group 1 had CVD and group 2 did not). Blood samples were stained with monoclonal antibodies (mAb) using Becton Dickinson (BD) reagents for the staining and binding of surface receptors CD4+, CD8+, CD14+, CD19+, CD16+, CD56+, CD59+, CD95+, and HLA DR+ and intracellular receptors TNF, IL-10, GM-CSF, VEGFR-2, IGF, and perforin. In addition, the following parameters were studied: questionnaire data (gender, age, alcohol consumption, smoking, physical activity, and marital status), clinical data (blood pressure (BP), heart rate (HR), body mass index (BMI)), comorbid conditions, and cardiovascular diseases (coronary heart disease (CHD), chronic heart failure (CHF), arterial hypertension (AH), previous myocardial infarction (PICS), diabetes mellitus (DM), atrial fibrillation (AF), and stroke). **Results:** The older patients with cardiovascular pathology had high levels of monocytes CD14+ (*p* = 0.014), low levels of CD8+ lymphocytes (*p* = 0.046), and low intracellular production of GM-CSF (*p* = 0.013) compared to the older people without CVD. **Conclusions:** The revealed differences in the expression of CD14+ monocytes indicate their role in the development of cardiovascular pathology associated with age-related changes. A decrease in cytotoxic CD8+ lymphocytes and intracellular GM-CSF production leads to an increased risk of developing cardiovascular diseases in older individuals. These observed changes with age will not only expand existing knowledge about the aging of the regulatory link of the immune system but also help to obtain data to predict CVD in older people. Thus, the obtained results support the use of these immunological markers to identify the risk of circulatory disease and a personalized approach in geriatric practice.

## 1. Introduction

With increasing life expectancy and aging of the population, there is a growing interest in understanding the aging process worldwide. With aging, morbidity and mortality from major age-related pathologies increase, primarily from cardiovascular pathology. Regardless of significant advances in understanding their pathogenesis and the introduction of modern therapeutic methods, cardiovascular diseases consistently occupy leading places in terms of population mortality [1,2,3]. Studies to identify biomarkers associated with the aging process are becoming key aspects of developing strategies for the prevention of age-associated diseases and improving the quality of life of the elderly. Studies show that the main cause of the development of cardiovascular diseases in the elderly is activation of the inflammatory response and the development of inflammatory aging (inflammaging) [3,4,5]. At the same time, one of the key signs of aging is a decrease in the functioning of the immune system, which makes the elderly more susceptible to infections, chronic inflammatory diseases, and vaccination failures [6]. Thus, immunoinflammation is a condition associated with an increase in the level of pro-inflammatory mediators, which gradually develops as a result of constant antigenic stimulation in older people. This antigenic stimulation can be caused by pathogens or cellular processes at the molecular level [7,8,9,10].

In the process of normal aging, and to a greater extent pathological aging, the subpopulation profile of peripheral blood lymphocytes, the thymus-dependent link of the immune system, which includes both the thymus itself and the composition and functionality of T- and B-cells, changes most significantly [7,8,9,10]. T-lymphocytes, as the main participants in the cellular link of the immune system, play an important role in the early stages of atherosclerosis development, since T-cells, both CD4+ and CD8+, are found inside atheromatous plaque [11,12]. A study of subpopulations of CD4+ and CD8+ lymphocytes demonstrated their increased level in middle-aged patients with CVD [12]. Further studies aim to identify the prognostic significance of these markers, including in older people.

In the last decade, the roles of CD14 monocytes and their subtypes in the risk of developing heart failure, proatherosclerotic activity, and contributions to the progression of cardiac ischemia have also been actively studied [13,14,15]. B-lymphocytes (CD19) are also involved in the process of modulating atherosclerosis, promoting myocardial remodeling and fibrosis, which subsequently worsens heart function [16,17]. There have been several studies on the role of CD56+ (NK lymphocytes) cells in the development of atherosclerosis, coronary heart disease, and ACS, but their exact participation in the development of CVD in older people has not been proven. Additionally, the effect on cardiac remodeling is currently being studied [18,19]. Scientific research shows that activation of the complement system significantly increases ischemic tissue damage in experimental myocardial infarction as a result of the direct effect of the membrane lysing complex on cardiomyocytes [20]. The inhibitor of this complex, protectin (CD59), is significantly expressed in the normal membrane of cardiomyocytes, but its content decreases in the infarction zone, a result of which the cardiac tissue becomes sensitive to the effects of the membranolytic complex [21]. These studies have shown the role of natural killers, monocytes, B-lymphocytes, and the complement system in the development and progression of the atherosclerotic process. However, further study and determination of the prognostic role of these markers in the process of cardiovascular aging are required.

In the inflammatory processes accompanying aging, the imbalance of cytokine regulation is of particular importance [22,23]. An increased level of tumor necrosis factor α (TNF-α) and a decreased level of interleukin 10 (IL-10) in the blood serum of elderly people are directly associated with various diseases, disability, and death [24,25]. Vascular endothelial growth factors (VEGF) and insulin-like growth factors (IGF) are also pathogenetically significant in the development of cardiac ischemia. They are potentially involved in the formation of the arterial bed (including the coronary arteries) and the development of collateral circulation. They correlate differently with many age-associated diseases, including diabetes mellitus, cancer, and cardiovascular diseases [26,27]. The presence of IGF receptors in the myocardium proves that growth hormone has both a direct and indirect effect on the regulation of the number and functional activity of cardiomyocytes, thus controlling the growth and contractile function of the myocardium [27]. In studies of patients with maladaptive post-infarction left ventricular remodeling, higher levels of GM-CSF were noted in the early and late post-infarction periods. GM-CSF has various biological effects: it activates cell proliferation, enhances chemotaxis and adhesion, stimulates the production of pro-inflammatory factors, and improves cell phagocytosis and antigen presentation [28,29,30]. The role of cytokines in the development of cardiovascular diseases is currently well studied, but research on the role of cytokines in the aging process and their prognostic value is ongoing.

Analyzing the immunological profile of blood lymphocytes and the intracellular production of cytokines involved in inflammation processes will allow us to identify a number of biomarkers closely associated with cardiovascular inflammation. These biomarkers can serve not only as indicators for the early diagnosis of CVD but also as potential therapeutic targets. In this regard, the aim of this study was to identify immunological markers associated with the process of inflammatory aging in older individuals with cardiovascular diseases.

## 2. Materials and Methods

### 2.1. Subjects

In accordance with the aim of this study, elderly patients were recruited for 5 months from 5 May to 30 September 2024 in clinics and medical centers of Almaty according to the inclusion/exclusion criteria. This study included 52 people over 60 years of age (group 1 (30 participants) had cardiovascular disease, and group 2 (22 people) did not). There were 18 men with an average age of 82.9 ± 10.0 years, and 34 women with an average age of 81.5 ± 10.0 years. All participants were provided with complete, reliable, and objective information about the course of this study. All study participants provided informed consent.

Inclusion criteria: men and women over 60 years of age.

Exclusion criteria: HIV infection, known tuberculosis, acute infectious diseases within 3 months before inclusion, mental illnesses that limit adequate cooperation, diagnosed allergic reaction of any type, refusal to participate in this study.

The following indicators were taken into account for all participants: questionnaire data (gender, age, alcohol consumption, smoking, physical activity, marital status), clinical data (blood pressure (BP), heart rate (HR), body mass index (BMI)), comorbid conditions, and cardiovascular diseases (coronary heart disease (CHD), chronic heart failure (CHF), arterial hypertension (AH), previous myocardial infarction (PICS), diabetes mellitus (DM), atrial fibrillation (AF), and stroke). To identify comorbid conditions and heart diseases, an analysis of medical records (medical history, medical records, extracts) was performed.

### 2.2. Ethics Approval

This study was approved by the Local Ethical Committee of Al Farabi Kazakh National University (IRB00010790, approval number IRB-A515 from 9 November 2023). All participants provided written informed consent for the use of biomaterials in this study.

### 2.3. Sample Processing

Blood for immunological studies was taken in the morning on an empty stomach in a volume of 5 mL in a special lilac test tube with EDTA and sent to the immunology laboratory of the Scientific Centre for Obstetrics, Gynaecology and Perinatology (Almaty, Kazakhstan).

### 2.4. Immunophenotyping

Blood samples were stained with monoclonal antibodies (mAb) using Becton Dickinson (BD, Hungary Kft., BD Biosciences, Franklin Lakes, NJ, USA) reagents according to the manufacturer’s protocol (www.bdbiosciences.com).

#### 2.4.1. Multicolor Staining for Cell Surface Antigens and Intracellular Cytokines

Surface staining was performed on whole blood using the following anti-human fluorochrome-conjugated antibodies for 20 min in the dark at 4 °C, followed by red blood cells lysis with BD FACS™ lysing solution (BD Biosciences). The antibodies used were anti-CD4 fluorescein isothiocyanate (FITC) (clone RPA-T4), anti-CD8 FITC (clone RPA-T8), anti-CD14 FITC (clone M5E2), anti-CD16 FITC (clone 3G8), anti-CD56 FITC (clone B159), anti-CD19 FITC (clone HIB19), anti-CD59 phycoerythrin (PE) (clone p282 (H19)), anti-CD95 PE (clone DX2), and anti-HLA-DR peridinin chlorophyll protein (PerCP) (clone L243). Permeabilization of membranes with BD Cytofix/Cytoperm™ Plus Fixation/Permeabilization Kit (with BD GolgiStop™ protein transport inhibitor containing monensin) (Cat. No. 554715) was also carried out, followed by the introduction of mAb for staining and binding of intracellular receptors—anti-TNF PerCP-Cy5.5 (clone Mab11), anti-IL-10 PE (clone JES3-19F1), anti-GM-CSF PE (clone BVD2-21C11), anti-VEGFR-2 PE (CD309, clone 89106), anti-IGF PE (CD221, clone 1H7), and anti-Perforin PerCP-Cy5.5 (clone σG9), according to the BD Biosciences intracellular cytokine staining protocols.

#### 2.4.2. Flow Cytometry Analysis

For proper flow cytometry analysis, cells were examined using light microscopy to confirm that they are well dispersed. The total population of leukocyte cells was isolated using the CD45+ marker; then, lymphocytes and monocytes were isolated from this fraction. Concentration-matched isotype controls were used to set the gates and single-fluorochrome stained controls were used to compensate for spectral overlap. The immunophenotyping with cytokine production of cells was evaluated on a BD FACS CALIBUR flow cytometer (USA) and the data were analyzed using the CELLQuest program.

### 2.5. Statistical Analysis

Statistical analysis of the obtained data was performed using the SPSS Statistics 22.0 package (IBM, Armonk, NY, USA). The normality of the sample distribution was checked using the Shapiro–Wilk test. In the case of a normal distribution, the chi-square was used, and in the case of an abnormal distribution, the nonparametric Mann–Whitney U-test with Bonferroni correction was used. Since the sample size was small, the *p*-value was adjusted to *p* < 0.025 for multiple comparisons (*p* < 0.05 was divided by 2). Differences between the compared groups were considered statistically significant at *p* < 0.05. Correlation between variables was estimated using the Spearman correlation coefficient (r) with interpretation 0 to 0.3 being very weak, 0.3 to 0.5—weak, 0.5 to 0.7—average, 0.7 to 0.9—high, and 0.9 to 1—very high.

## 3. Results

### 3.1. Clinical Characteristics of the Study Groups

Comparative demographic and clinical characteristics of patients in both groups are presented in Table 1 and Table 2.

This study involved 52 people aged 60 years and older, of whom 18 (35%) were men and 34 (65%) were women, who were observed in Almaty clinics. The average age of men was 82.9 ± 10.0 years, and that of women was 81.5 ± 10.0 years. As can be seen from Table 1, in the group with CVD, patients were more often male, of Kazakh nationality, and married.

Comparative clinical characteristics of patients in both groups are presented in Table 2; in this case, qualitative indicators are shown as percentages (%) and quantitative ones as mean values (±).

In the group with CVD, such risk factors as smoking (*p* = 0.011) and overweight (*p* < 0.001) were statistically more often observed. Smoking is the main risk factor for the development of CVD and other diseases such as COPD, lung cancer, etc. Among people without CVD, a significantly more normal BMI was noted compared to the main group. Systolic blood pressure continuously increases with age, and is also a strong and independent risk factor for the development of cardiovascular diseases, and shows a reliable difference (*p* < 0.001). Heart rate, being an indicator of sympathetic reactivity, is a reliable and significant risk factor for the development of cardiovascular diseases and their complications (*p* = 0.040). For other risk factors, such as alcohol abuse and physical inactivity, no statistical difference was found between the groups.

Comorbidity. In the comorbidity analysis, concomitant diseases were identified for each subject and the percentage value is shown. On average, at least two diseases were encountered, and most often these are diseases of the musculoskeletal system and lungs.

Bronchial asthma, stroke, and cerebrovascular insufficiency were statistically more frequently detected in patients with CVD (*p* < 0.05). There was a tendency for the incidence of cancer, osteoarthritis, and cerebrovascular diseases to increase in group 1, but there were no significant differences.

**Figure 1:**
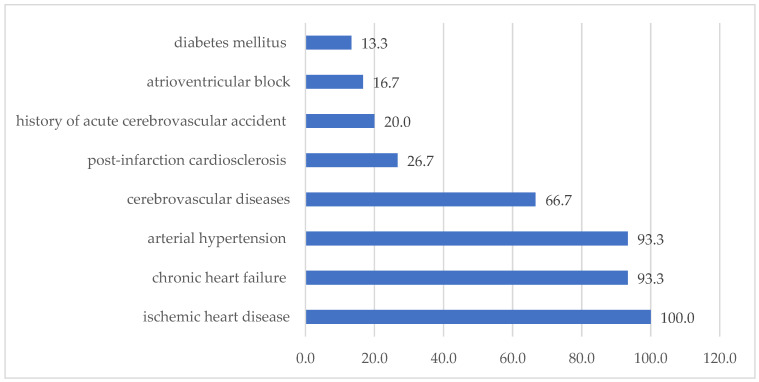
Structure of cardiovascular pathology in patients of group 1 (%).

The structure of cardiovascular pathology in group 1 is presented as follows (Figure 1).

All patients had ischemic heart disease (100%), chronic heart failure and arterial hypertension (93.3%), post-infarction cardiosclerosis (26.6%), diabetes mellitus (13.3%), and atrioventricular block (16.6%), and a history of acute cerebrovascular accident (20.0%) and cerebrovascular diseases (66.7%) was also taken into account.

### 3.2. Immunophenotyping of Blood Lymphocytes

Data on immunophenotyping of blood lymphocytes in the study groups are presented as medians and interquartile ranges (Table 3).

The conducted clinical and laboratory study revealed an increase in the level of CD14+ monocytes in elderly patients with cardiovascular pathology compared to its level in healthy individuals in the same age category (*p* = 0.014). At the same time, in elderly patients with cardiovascular pathology, a decrease in the level of CD8+ lymphocytes was revealed (*p* = 0.046). Generally, there was an increase in the expression of surface receptors CD16+, CD56+, CD19+, CD59+, and CD95+, but it did not show a reliable difference.

#### Intracellular Cytokines

In the group of older patients with CVD, a decrease in the intracellular production of GM-CSF was noted (*p* = 0.013) compared to the group without CVD. The level of intracellular production of other cytokines was generally higher than the reference values, but no statistically significant differences were found.

At the next stage, a correlation analysis was carried out between the identified statistically significant immunological indicators and clinical data. Correlation analysis revealed a weak relationship between CD14+ and body mass index (BMI) (r = 0.337, *p* < 0.05); between CD14+ and post-infarction cardiosclerosis (PICS) in patients with CVD (r = 0.329, *p* < 0.05); between HLA-DR+, Perforin+, and IGF+ and BMI (r = 0.375, r = 0.345, r = 0.424, respectively, *p* < 0.05); and between TNF and diastolic blood pressure (DBP), IGF, and HR (r = 0.429, *p* < 0.05).

Building upon our previous research, where machine learning was successfully applied to immunological markers (CD59, CD16, TNF-α) for stratifying the risk of premature cardiovascular aging [31], this study implements an optimized XGBoost model. Fine-tuning of the algorithm yielded high performance metrics, namely, 98.5% accuracy, 99.14% precision, 98.29% recall, and a 98.71% F1-score, confirming its reliability and potential for integration into clinical decision support systems.

## 4. Discussion

In recent decades, various theories of aging have been hotly debated. The immunological theory of aging is based on the deterioration of the immune system (immunoaging), which consists of structural and functional changes in the adaptive and innate immune systems [3,4,5]. With aging, immunodeficiency of all components of the immune system develops. This includes a decrease in the number of T-lymphocytes, a decrease in the diversity of the antigen repertoire, and disruption of the immune system in relation to its own tissues, which ultimately leads to increased susceptibility to infections and malignant tumors and an increase in age-associated diseases. However, the rate of development of such a scenario depends on the background against which these changes occur. Whether the inflammatory phenotype is a reflection of healthy aging or is associated with pathological processes in the body, primarily cardiovascular diseases, remains to be determined.

In order to identify immunological markers associated with cardiovascular diseases in elderly patients, in comparison with healthy elderly people, we studied an extended panel of immunological parameters, including links of both innate (monocytes, natural killers, and activation markers) and adaptive immunity (regulatory T-lymphocytes CD4+ and CD8+), as well as the intracellular production of pro- and anti-inflammatory cytokines and growth factors. In the current study, it was revealed that in pathological aging associated with cardiovascular diseases, activation of the innate immunity component CD14+ monocytes (*p* = 0.014), a decrease in the adaptive link between the immune system and cytotoxic T-lymphocytes CD8+ (*p* = 0.046), and a decrease in the intracellular production of GM-CSF by lymphocytes (*p* = 0.013) are of great importance. In the current study, elderly patients with cardiovascular diseases showed a tendency towards increased expression of surface receptors CD16+, CD56+, and CD59+ on blood lymphocytes, but no statistically significant differences with the control group were found.

Our study is consistent with the literature data on the role of monocytes in the development of cardiovascular pathology. The proatherosclerotic activity of CD14+ monocytes has been shown during the progression of coronary heart disease [14,32].

CD14+ monocytes are a key component of innate immunity and participate in inflammatory reactions, including the response to vascular wall damage. According to the classification, monocytes are divided into three subpopulations: “classic” CD14++CD16- have high phagocytosis and cytotoxicity and provide antimicrobial and anticarcinogenic protection; “intermediate” CD14++CD16+ (“inflammatory”) produce the most powerful pro-inflammatory factor α-TNF and generate active oxygen radicals; “non-classical” CD14+CD16++ (“patrolling”) enhance the processes of regeneration and repair of damaged tissues and the synthesis of anti-inflammatory cytokines and growth factors [33]. T-lymphocytes (T-cells) were identified as important regulators of endothelial function in mouse models, and pro-inflammatory and senescent T-cell subsets were associated with endothelial dysfunction in middle-aged adults with hypertension [34,35]. Unlike elderly patients with cardiovascular pathology, middle-aged patients with cardiovascular pathology have a more diverse range of changes in their immunological parameters. It has been shown that adverse effects of coronary heart disease can be caused by an increase in the level of CD56+ (NK cells) in the peripheral blood [12,16]. The activation of CD95+ apoptosis markers contributes to the death of cardiomyocytes and an increase in the infarction zone [12]. According to the latest data, CD59+ affects the development of atherosclerosis and cardiac ischemia. There is also scientific evidence that the protein can protect cells from this damage [19]. Deficiency of the pore-forming protein perforin can lead to weight gain and obesity and insulin resistance when using high-fat diets [23].

Clinical and experimental studies conducted over the past decade have indicated an important role of pro-inflammatory cytokines (TNF-α) in the development of atherosclerosis and the occurrence of cardiovascular dysfunction in arterial hypertension and pathological myocardial remodeling, including coronary heart disease [24]. Both decreased and increased levels of anti-inflammatory cytokine (IL-10) play roles in the risk of developing cardiovascular diseases [25]. It has been shown that IGF (insulin-like growth factor) deficiency leads to an increase in the thickness of the intimal complex, which is a predictor of the development of atherosclerosis of peripheral and coronary arteries. Additionally, the introduction of IGF helps to improve cardiac parameters in patients with cardiomyopathy, coronary heart disease, and heart failure [27]. Vascular endothelial growth factor VEGFR-2 plays a role in the development of cardiovascular diseases [25].

Vascular endothelial growth factor VEGFR-2 increases the risk of developing cardiovascular disease, but it also plays a role in the development of new blood vessels in the heart after myocardial infarction [25]. The stimulation of VEGFR-2 may promote heart muscle recovery [26]. In addition, the introduction of GM-CSF for 4–5 days led to a decrease in the size of the ischemic damage zone and an improvement in cardiac function, while higher levels of GM-CSF were found in patients with maladaptive post-infarction remodeling of the left ventricle [28,29]. A decrease in this factor increases the risk of cardiovascular diseases.

This study identified a number of biomarkers closely associated with cardiovascular inflammation in elderly patients. It was found that cardiovascular risk in elderly patients is associated with the activation of classical monocytes with pro-inflammatory potential and the suppression of CD8+ suppressor lymphocytes and a decrease in the intracellular production of the growth factor GM-CSF.

With the development of immunomics technology, we have new tools for comprehensively identifying the relationship between cardiovascular diseases and immune dysfunctions, primarily associated with the aging process of the immune system [36]. Recent studies have highlighted the potential for identifying early biomarkers of cardiovascular pathology based on knowledge of the disease mechanisms and having significance for the development of methods not only for diagnosis but also for the prediction of CVD [37].

Our study had limitations that need to be considered in future studies. Further studies will focus on different monocyte populations that reflect different functional states. For risk stratification in subclinical CVD, studies on the relationship between the activation of pro-inflammatory monocytes and the determination of global longitudinal deformation using the speckle tracking method are also needed. This may contribute to the understanding of common pathogenetic mechanisms in CVD in the elderly [37]. The identification of new markers will provide a personalized approach to the diagnosis and prognosis of cardiovascular diseases, and will also enable the use of artificial intelligence methods, including machine learning and big data analytics, to calculate individual risk [31,38].

## 5. Conclusions

The revealed differences in the expression of CD8+ cytotoxic lymphocytes and CD14+ monocytes indicate their role in the development of cardiovascular aging associated with age-associated changes. A decrease in intracellular GM-CSF production leads to an increased risk of developing cardiovascular diseases in older individuals. These changes with age will not only expand existing knowledge about the aging of the regulatory link of the immune system but also help to obtain data to predict CVD in older people. The obtained results support the use of these immunological markers to identify the risk of circulatory disease and a personalized approach in geriatric practice.

## Figures and Tables

**Table 1:** Demographic characteristics of patients in the study groups.

Characteristic	with CVD (abs, %), n = 30	Without CVD (abs, %), n = 22	*p* Value
Man	13 (43.3%)	5 (22.7%) *	0.004
Woman	17 (77.3%)	17 (56.7%)	0.845
Married	17 (56.7%)	9 (40.9%) *	0.002
Widow/widower	11 (36.7%)	12 (54.5%) *	0.003
Kazakhs	20 (66.7%)	9 (40.9%) *	0.005
Russian	7 (23.3%)	9 (40.9%)	0.647
Uigur	1 (3.3%)	0 (0.0%)	0.011
Other	2 (6.7%)	4 (18.2%)	
Disability: Yes	6 (20.0%)	4 (18.2%)	
Disability: No	24 (80.0%)	18 (81.8%)	

* differences between groups (*p* < 0.05).

**Table 2:** Clinical characteristics of patients in the study groups.

Characteristic	with CVD (abs, %), n = 30	Without CVD (abs, %), n = 22	*p* Value
Smoking (abs, %)	7 (23.3%)	4 (18.2%)	0.011 *
Alcohol abuse (abs, %)	2 (6.7%)	4 (18.2%)	0.714
Body mass index (kg/m^2^: M ± m)	25.7 ± 0.7 0	22.0 ± 0.51	0.000 *
Low physical activity < 30 min (M ± m)	12.5 ± 1.75	20.06 ± 12.7	0.358
Systolic blood pressure (M ± m)	136.33 ± 2.1	126.14 ± 1.5	0.000 *
Diastolic blood pressure (M ± m)	81.17 ± 1.5	80.23 ± 1.6	0.831
Heart rate (M ± m)	81.40 ± 1.4	76.86 ± 1.2	0.040 *
**Comorbidity**			
Bronchial asthma	1 (3.3%)	0 (0%)	0.000 *
Chronic obstructive pulmonary disease	9 (30.0%)	10 (45.5%)	0.394
Oncopathology	3 (10.0%)	1 (0%)	0.354
Stroke	6 (20.0%)	0 (0%)	0.043 *
Osteoarthritis	18 (60.0%)	15 (68.2%)	0.754
Iron deficiency anemia	3 (10.0%)	4 (18.2%)	0.658
Cerebrovascular diseases	20 (66.7%)	12 (54.5%)	0.549
Discirculatory encephalopathy	23 (76.7%)	9 (40.9%)	0.020 *
Chronic renal failure	8 (26.7%)	3 (13.6%)	0.428

Note. Data presented as n (%). Chi-square test; * differences between groups (*p* < 0.05).

**Table 3:** Immunophenotyping of blood lymphocytes in the study groups.

Characteristic	Group 1 with CVD (n = 30)		Group 2 without CVD (n = 22)		*p*
	Me	IQR	Me	IQR	
Subpopulation peripheral blood lymphocyte profile (surface receptor markers)					
CD4+	34.57	31.59–42.77	36.77	32.84–41.8	0.578
CD8+	27.26	25.56–31.41	30.23	27.93–30.92	0.046 *
CD14+	4.08	2.68–4.91	3.02	2.46–3.83	0.014 *
CD19+	12.93	11–16.09	11.97	11.43–16.27	0.978
CD16+	31.36	27.54–33.6	30.59	25.08–33.63	0.448
CD56+	90.31	81.58–96.34	82.82	78.63–94.81	0.232
CD59+	90.30	79.02–96.17	82.82	80.82–91.18	0.232
HLA-DR+	24.0	21.62–31.04	24.6	22.15–28.42	0.553
CD95+	71.46	55.63–77.84	70.23	57.79–81.33	0.963
Intracellular production of cytokines and growth factors					
TNF	3.88	2.49–5.86	4.68	3.16–6.07	0.195
IL-10	2.14	1.66–2.57	1.9	1.58–2.47	0.295
Perforin	4,7350	3.01–5.1	4355	3.52–4.59	0.259
IGF	86.39	80.13–92.74	80.3	78.65–90.01	0.122
VEGF2	72.05	66.87–74.84	69.09	65.94–72.92	0.195
GM-CSF	1.63	0.96–2.32	2.32	1.7–3.5	0.013 *

Note. Data presented as Me [IQR]. Mann–Whitney U-test; * differences between groups (*p* < 0.05), adjusted to *p* < 0.025.

## Data Availability

Data are unavailable due to privacy or ethical restrictions.
